# Multi-Scale Guided Context-Aware Transformer for Remote Sensing Building Extraction

**DOI:** 10.3390/s25175356

**Published:** 2025-08-29

**Authors:** Mengxuan Yu, Jiepan Li, Wei He

**Affiliations:** 1 School of Remote Sensing and Information Engineering, Wuhan University, Wuhan 430079, China; mengxuanyu@whu.edu.cn; 2 State Key Laboratory of Information Engineering in Surveying, Mapping and Remote Sensing, Wuhan University, Wuhan 430079, China

**Keywords:** building extraction, remote sensing, deep learning, window attention mechanism

## Abstract

Building extraction from high-resolution remote sensing imagery is critical for urban planning and disaster management, yet remains challenging due to significant intra-class variability in architectural styles and multi-scale distribution patterns of buildings. To address these limitations, we propose the Multi-Scale Guided Context-Aware Network (MSGCANet), a Transformer-based multi-scale guided context-aware network. Our framework integrates a Contextual Exploration Module (CEM) that synergizes asymmetric and progressive dilated convolutions to hierarchically expand receptive fields, enhancing discriminability for dense building features. We further design a Window-Guided Multi-Scale Attention Mechanism (WGMSAM) to dynamically establish cross-scale spatial dependencies through adaptive window partitioning, enabling precise fusion of local geometric details and global contextual semantics. Additionally, a cross-level Transformer decoder leverages deformable convolutions for spatially adaptive feature alignment and joint channel-spatial modeling. Experimental results show that MSGCANet achieves IoU values of 75.47%, 91.53%, and 83.10%, and F1-scores of 86.03%, 95.59%, and 90.78% on the Massachusetts, WHU, and Inria datasets, respectively, demonstrating robust performance across these datasets.

## 1. Introduction

The identification and localization of buildings constitute a fundamental task in regional planning, carrying significant implications for urban planning [1] and demographic analysis [2]. This geospatial capability provides critical support for governmental decision-making in urban planning while delivering essential datasets for urban environmental monitoring [3], disaster risk management [4], and sustainable urban development initiatives [5].

Although buildings in remote sensing imagery exhibit significant spatial distribution and spectral feature variations, early research primarily focused on two universal identifiable characteristics of their geometric morphology: straight edges and shadow casting [6]. Specifically, edge features have been effectively utilized through image processing techniques such as edge detection [7] and Hough transform [8], enabling accurate extraction of geometric boundaries for regular buildings. Meanwhile, shadow features [9,10] have been incorporated as crucial auxiliary discriminant features, substantially improving building extraction accuracy. With modern societal development, high-rise buildings demonstrate increasing complexity and diversity in both morphological configurations and material compositions, presenting distinct functional advantages over traditional low-rise structures. However, conventional edge and shadow feature extraction methods suffer from significant performance degradation due to the difficulty in constructing robust and universal modeling frameworks capable of adapting to such complexity [11], which poses new challenges to existing technical systems.

To address these limitations, deep-learning-based methods have emerged as highly promising solutions [12]. Conventional convolutional neural networks (CNNs) [13] demonstrate remarkable capabilities in local feature extraction through their hierarchical learning architecture. However, the fully connected layers at the final stage of the architecture require fixed-size input dimensions, which introduces inherent limitations when processing building extraction tasks characterized by significant scale variations and morphological diversity [14,15].

The Fully Convolutional Network (FCN), proposed by Shelhamer et al. [16], is specifically designed for semantic segmentation tasks. In contrast to traditional CNNs, FCNs can process input images of arbitrary sizes and generate corresponding prediction masks with matched spatial dimensions, thereby significantly enhancing the preservation of spatial information in end-to-end pixel-wise prediction tasks. The core architecture employs an encoder–decoder framework, where the encoder is typically constructed from classical backbone networks (e.g., VGG [15], ResNet [17], Res2Net [18], and PVTv2 [19]), generating high-dimensional, low-resolution feature representations through multi-level feature extraction. The decoder progressively restores spatial resolution via upsampling operations, remapping high-level semantic encodings back to the pixel space to achieve end-to-end fully convolutional semantic segmentation [20]. Currently, researchers have proposed various innovative decoding architectures. Several studies [21,22,23,24,25,26,27,28,29] enrich feature representations by aggregating multi-scale contextual information to capture building-related cues, while others [30,31,32,33,34,35,36] employ channel-wise or spatial attention mechanisms for dynamic feature calibration. Additionally, some works [37,38,39,40,41,42,43,44] incorporate boundary supervision during the decoding phase to refine segmentation performance. Furthermore, cascade-style decoding frameworks [45,46,47,48,49,50,51] adopt coarse-to-fine progressive refinement strategies, systematically improving prediction accuracy through multi-stage decoding processes.

**Figure 1:**
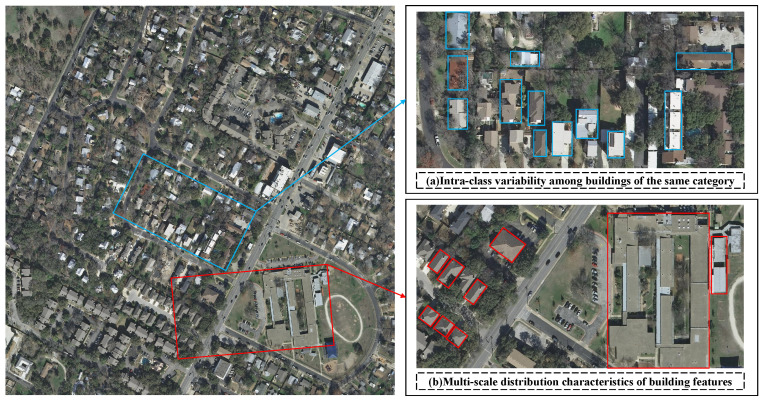
Challenges confronting conventional decoder mechanisms: (**a**) intra-class variability among buildings of the same category (highlighted by blue bounding boxes); (**b**) multi-scale distribution characteristics of building features (highlighted by red bounding boxes).

Although FCN-based high-resolution remote sensing building extraction has achieved significant progress, notable style variations among different buildings (as illustrated by the blue bounding boxes in Figure 1, where roof colors and shapes exhibit substantial differences) lead to markedly divergent edge features and geometric structures of buildings within the same image, making unified modeling challenging for traditional edge detection methods. Furthermore, modern architectures employ diverse construction materials, resulting in significant variations in material properties and spectral-spatial characteristics, all of which contribute to pronounced intra-class variability among buildings of the same category. Taking the buildings highlighted by red bounding boxes in Figure 1 as examples, building scales span a wide range from tens or hundreds of pixels to thousands, demonstrating systematic multi-scale distribution patterns. While current decoding mechanisms have proposed respective solutions addressing either intra-class variability or multi-scale characteristics, most approaches focus exclusively on one aspect. Recent CNN–Transformer hybrid architectures [52,53,54,55] tackle intra-class variability and multi-scale distribution. However, their fixed multi-scale design cannot adapt to the diverse building scales, and spatial misalignment, together with semantic gaps, still hinders effective feature fusion. These limitations lead to systematic errors, including the omission of small-scale buildings and material-related misclassifications, which become particularly evident when processing modern architectural clusters characterized by morphological diversity and large-scale variations.

To address these challenges, we propose a multi-scale guided context-aware network based on the Transformer architecture, termed Multi-Scale Guided Context-Aware Network (MSGCANet). Our network first employs a pretrained Pyramid Vision Transformer (PVTv2) [19] to generate multi-level feature maps. Building upon this foundation, we innovatively propose the following: (1) Contextual Exploration Module: By synergizing asymmetric convolutions with progressive dilated convolutions, this module establishes hierarchical contextual representations through a multi-scale receptive field expansion mechanism, generating enhanced context-aware features. (2) Window-Guided Multi-Scale Attention Mechanism: This mechanism explicitly establishes cross-scale spatial feature dependencies through a dynamic window partitioning strategy, enabling dynamic feature fusion while preserving local structural details and enhancing global contextual awareness. (3) The cross-level Transformer decoder: As a cross-level feature integration decoder, it performs unified alignment of multi-level semantic features and jointly conducts channel-spatial modeling, effectively achieving dual enhancement of both semantic representation and detail preservation. This framework ultimately yields refined building extraction results.

The proposed MSGCANet significantly enhances multi-scale feature modeling capabilities while achieving synergistic optimization of semantic understanding and geometric details. The key contributions of this work include the following:

- A hierarchical receptive field expansion mechanism based on asymmetric convolutions and progressive dilated convolutions is proposed, which synergistically integrates dynamic multi-scale feature fusion with residual-guided optimization to significantly enhance contextual modeling capabilities in dense prediction tasks;
- We propose a dynamic multi-scale feature fusion method using hierarchical attention for adaptive cross-scale aggregation, preserving local geometry and balancing global–local contexts. Combined into a Transformer-based decoder, it employs deformable convolutions for spatially adaptive feature alignment;
- Demonstration of refined building extraction performance on multiple public datasets [56,57,58], with significant improvements in evaluation metrics, validating the superiority of our proposed MSGCANet.

The remainder of this paper is organized as follows. Section 2 presents a comprehensive review of related work on semantic segmentation decoder mechanisms. Section 3 provides a detailed analysis and introduction of the proposed MSGCANet architecture and its components. Section 4 presents the experimental setup and results analysis. Section 5 conducts ablation studies on the proposed modules. Finally, Section 6 concludes the paper.

## 2. Related Work

The innovation of the proposed MSGCANet primarily lies in its decoding strategy design. Therefore, this section systematically reviews the evolutionary trajectory and technical limitations of existing decoding methods. Existing decoder methods can be primarily categorized into the following types:

(1) Multi-scale context-based methods: These approaches [21,22,23,24,25,26,27,28] construct multi-scale context modules to characterize foreground objects with varying scales and appearances. The ASPP module proposed in DeepLab [21] achieves multi-scale context capture in a single forward pass through parallel multi-branch architectures and dynamic receptive field adjustment. Subsequently, Zhao et al. [22] developed the Pyramid Pooling Module (PPM), which for the first time realized unified modeling of global and local contexts. Chen et al. [23] innovatively integrates atrous convolution with pooling operations in a four-branch parallel architecture, while another research direction [24,25] introduces graph neural network frameworks that map multi-scale features to graph nodes and model cross-scale dependencies via attention mechanisms. State-of-the-art approaches [26,27,28] have successfully addressed the persistent trade-off between fine-grained semantic understanding and geometric precision in conventional methods through innovative cross-domain feature interaction mechanisms. A paradigmatic example is the dual-attention selective kernel network developed by Sultonov et al. [28], which achieves feature refinement via adaptive multiscale feature fusion integrated with cascaded channel-spatial attention mechanisms.

(2) Attention-based methods: These approaches [30,31,32,33,34,35,36] employ synergistic integration of self-attention and cross-attention mechanisms to model long-range dependencies for enhanced feature consistency. Several studies [30,31] have demonstrated that joint modeling of spatial attention (PAM) and channel attention (CAM) can effectively capture global dependencies. With further research development, some works [32,33] have integrated frequency-domain attention with multi-scale detection methods into attention mechanisms. The dual-path hierarchical attention framework proposed by Wang et al. [34] combines window-based linear self-attention for global context modeling with convolutional spatial detail preservation to achieve efficient building extraction from high-resolution remote sensing imagery. Current research focuses on task-specific lightweight attention mechanisms, as exemplified by the GCM+LCM module proposed by Zhai et al. [35] for UAV change detection tasks, which effectively captures bi-temporal differences through global–local contrastive mechanisms. Further advancing this direction, Fu et al. [36] developed a complementarity-aware fusion mechanism that explicitly decouples shared and distinct features between convolutional and Transformer branches, enforcing triplet constraints to maximize cross-branch feature interactions while adaptively exchanging local patterns and global dependencies through gated feature recalibration, thereby achieving dynamic fusion of local and global contextual representations.

(3) Boundary-supervised methods: These approaches [37,38,39,40,41,42,43,44] enhance the geometric precision of segmentation boundaries through boundary-supervised loss functions. The technical evolution exhibits three characteristic phases: (a) initial stage employing post-processing methods like CRF [37] for boundary refinement; (b) intermediate stage indirectly enhancing boundary representation via multi-scale feature fusion [38]; and (c) recent methodologies have effectively addressed core challenges in conventional approaches—including boundary ambiguity, small target omission, and complex background interference—through three pivotal innovations: optimization of loss functions [39,40], enhancement of boundary features via multimodal/multiscale feature interaction [41,42], and implementation of novel network architectures [43,44], collectively advancing segmentation precision.

(4) Coarse-to-fine methods: These approaches [45,46,47,48,49] employ multi-stage progressive optimization to progressively refine segmentation results from coarse predictions to sub-pixel boundary delineation. The framework proposed by Li et al. [45] pioneered difficulty-aware progressive optimization through hierarchical network partitioning, while the recursive framework by Jing et al. [46] further advanced multi-scale feature fusion. Researchers have also developed specialized networks for building extraction, including the boundary refinement strategy by Guo et al. [47], the ASPP-based multi-scale fusion architecture by Sheikh et al. [48], and the innovative dual-task coordination mechanism integrating vector extraction with semantic segmentation proposed by Liu et al. [49].

While existing decoder research has made significant progress, most approaches still rely on predefined parameter configurations, lacking dynamic scale adaptability and exhibiting insufficient alignment between low-level features and high-level semantics. Specifically, although multi-scale context methods capture multi-scale information through fixed modules, they struggle to dynamically adjust receptive fields or fusion strategies based on input features, resulting in inaccurate feature representation when handling buildings with substantial scale variations. Attention mechanisms, while capable of modeling long-range dependencies, overly depend on static weight configurations, frequently leading to spatial detail loss or computational redundancy during global feature integration. Boundary-supervised methods, despite incorporating loss function optimization, continue to face fundamental challenges, including boundary ambiguity, small target omission, and complex background interference, which constrain their ability to enhance building geometric precision. Furthermore, coarse-to-fine strategies suffer from error propagation in initial predictions that compromises final boundary refinement. These limitations collectively hinder decoders’ capacity to dynamically accommodate the cross-scale representation requirements of buildings, adversely affecting multi-level feature expression from microscopic details to macroscopic spatial layouts.

Moreover, existing methods predominantly address intra-class variations and multi-scale features in isolation, neglecting their synergistic effects. In recent years, researchers have proposed hybrid CNN-Transformer architectures as representative fusion approaches [52,53,54,55] that leverage multimodal feature complementarity, offering innovative solutions to simultaneously tackle both intra-class variability and multi-scale challenges. These methods employ local-global feature coordination and dynamic contextual modeling to jointly optimize local sensitivity and global consistency, thereby significantly mitigating the performance bottlenecks inherent in traditional single-perspective approaches. However, static multi-scale modeling struggles to adapt to the actual scale distribution of buildings, while spatial misalignment and semantic gaps during feature fusion constrain cross-modal synergistic effects. Additionally, the computational overhead of attention mechanisms limits their practical application to high-resolution imagery. These inherent limitations collectively result in constrained accuracy when processing modern architectural clusters characterized by morphological diversity and substantial scale variations.

## 3. Methodology

### 3.1. Overview

**Figure 2:**
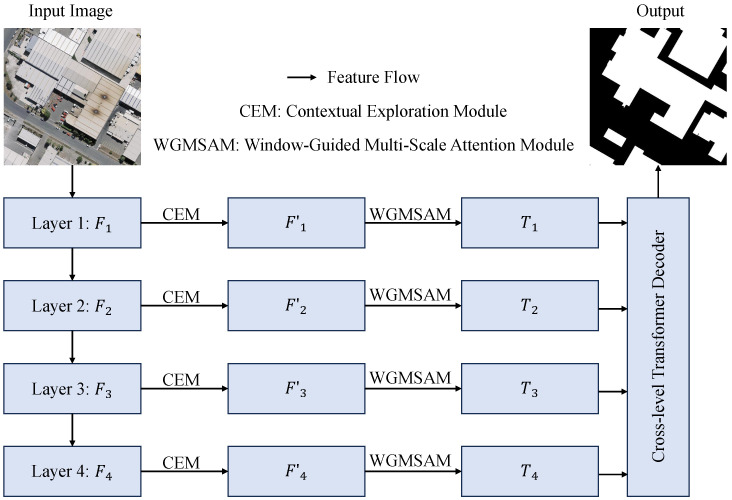
Architecture of Multi-Scale Guided Context-Aware Network (MSGCANet).

To tackle the challenges associated with building extraction from high-resolution remote sensing images, we propose MSGCANet, a multi-scale guided, context-aware Transformer architecture (Figure 2).

The network extracts hierarchical features ${\left\{F_{i}\right\}}_{i=1}^{4}$ at four distinct levels using the PVTv2 backbone network [19]. Each feature map $F_{i}$ is subsequently enhanced by the Contextual Exploration Module (CEM), which captures multi-scale contextual information through a parallel multi-branch architecture, ultimately producing refined features ${\left\{F_{i}^{\prime }\right\}}_{i=1}^{4}$. To address the intra-class variability and multi-scale distribution characteristics of building features in remote sensing imagery, MSGCANet innovatively designs a decoding mechanism: First, feature alignment is achieved, followed by element-wise averaging to generate the unified feature representation X that preserves hierarchical information. The integrated feature X is then processed by the Window-Guided Multi-Scale Attention Mechanism (WGMSAM) to obtain level-specific features ${\left\{T_{i}\right\}}_{i=1}^{4}$, which simultaneously capture local details and global contextual information while maintaining the structural integrity of buildings. These features are further integrated through a cross-layer Transformer decoder to produce the reconstructed feature $X_{\mathrm{attn}}$. The final building extraction prediction map P is generated through convolutional operations followed by upsampling.

### 3.2. Contextual Exploration Module

The PVTv2 backbone network [19] exhibits three critical issues in feature extraction: (1) high-level feature maps suffer from significant spatial resolution reduction due to repeated downsampling operations, leading to local detail loss and edge blurring; (2) simple upsampling or convolutional operations between different hierarchical feature maps lack effective cross-scale interaction mechanisms, resulting in insufficient fusion of semantic and spatial information; (3) the fixed-size window partitioning scheme significantly constrains cross-window global interaction in deep feature maps, thereby restricting receptive field expansion and consequently impairing comprehensive contextual information extraction from the feature representations.

**Figure 3:**
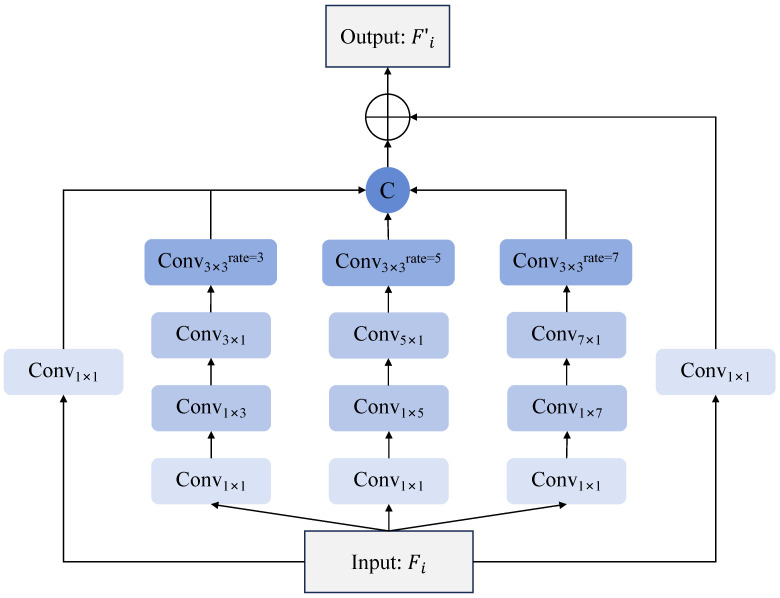
Structure of Contextual Exploration Module.

To reduce the complexity of subsequent decoding processes and achieve more precise predictions, we propose an innovative Contextual Exploration Module (Figure 3) to enhance multi-level feature representations. The module adopts a parallel multi-branch architecture that systematically constructs geometrically progressive receptive field expansion through its concurrent processing branches. This innovative design generates multi-scale overlapping contextual perception zones, enabling simultaneous extraction of local textural details, mid-range structural patterns, and global spatial relationships, which form a critical foundation for subsequent multi-scale window decoding operations.

Specifically, the input feature map $F_{i}\in R^{H\;\times \;W\;\times \;C_{in}}$ is processed through four independent paths:

1. The base path employs 1×1 pointwise convolution for channel transformation
  (1) $$Y_{0}={\mathrm{Conv}}_{1\;\times \;1}\;*\;F_{i}+b_{0}$$
  where ${\mathrm{Conv}}_{1\;\times \;1}$ transforms the channel dimension from $C_{in}$ to $C_{out}$. The output feature $Y_{0}\in R^{H\;\times \;W\;\times \;C_{out}}$ strictly maintains the original spatial resolution with a 1 × 1 pixel receptive field, specifically designed for capturing microscopic-scale features. Notably, this initial channel transformation convolution is consistently incorporated in all subsequent three branches, ensuring identical feature space foundations prior to further operations.
2. The primary expansion path adopts a four-stage cascade structure: 1 × 1 channel compression convolution, followed by 1 × 3 and 3 × 1 asymmetric convolution pairs, and finally a 3 × 3 dilated convolution with rate 3. This yields output feature $Y_{1}\in R^{H\;\times \;W\;\times \;C_{out}}$ with an effective 7 × 7 receptive field.
3. The intermediate expansion path extends the primary path by using 1 × 5 and 5 × 1 asymmetric convolution pairs combined with a rate-5 3 × 3 dilated convolution, producing output feature $Y_{2}\in R^{H\;\times \;W\;\times \;C_{out}}$ with a 19 × 19 receptive field.
4. The advanced expansion path further implements 1 × 7 and 7 × 1 asymmetric convolution combinations coupled with a rate-7 3 × 3 dilated convolution, expanding the receptive field of output feature $Y_{3}\in R^{H\;\times \;W\;\times \;C_{out}}$ to 31 × 31 pixels, specifically optimized for capturing global context of large-scale targets.

The branch outputs are concatenated sequentially from $Y_{0}$ to $Y_{3}$ along the channel dimension through a deterministic concatenation operation to construct a high-dimensional feature representation $Y_{cat}\in R^{H\;\times \;W\;\times \;4C_{out}}$, which mathematically manifests as a linear reorganization of channel indices:

(2) $$Y_{cat}\left(h,w,k\right)=\left\{\begin{array}{cc} Y_{0}\left(h,w,k\right), & \;0\leq k<C_{out}\\ Y_{1}\left(h,w,k-C_{out}\right), & \;C_{out}\leq k<2C_{out}\\ Y_{2}\left(h,w,k-2C_{out}\right), & \;2C_{out}\leq k<3C_{out}\\ Y_{3}\left(h,w,k-3C_{out}\right), & \;3C_{out}\leq k<4C_{out} \end{array}\right.$$

This concatenation operation preserves the spatial structure of each branch feature by stacking them linearly along the channel direction at (h,w) coordinates. The concatenated feature tensor is then fed into a learnable feature fusion layer, in which a 3 × 3 convolution first linearly combines the multi-branch features across all channels, followed by a ReLU activation that introduces non-linear mappings by suppressing negative responses and enhancing positive activations, allowing the module to capture richer cross-scale and cross-channel interactions.

(3) $$Y_{fused}={\mathrm{Conv}}_{3\;\times \;3}\left(Y_{cat}\right)$$

where ${\mathrm{Conv}}_{3\times 3}$ denotes the standard convolution operation with kernel size 3×3, and its weight parameters are $\Theta \in R^{3\;\times \;3\;\times \;4Cout\;\times \;C_{out}}$. Notably, the 3 × 3 convolution in the fusion layer not only compresses feature channels but, more importantly, establishes a dynamic weighting mechanism for cross-scale features, enabling adaptive contribution adjustment based on input content.

To maintain feature stability, we incorporate residual learning. The original input $F_{i}$ undergoes channel alignment via 1×1 convolution to produce residual feature $Y_{res}\in R^{H\;\times \;W\;\times \;C_{out}}$. The final enhanced feature $F_{i}^{\prime }$ is obtained by:

(4) $$F_{i}^{\prime }=\mathrm{ReLU}\left(Y_{fused}+Y_{res}\right)$$

The enhanced output features $F_{i}^{\prime }$ incorporate multi-scale contextual information ranging from local details to global semantics through a hierarchical contextual exploration mechanism, while systematically preserving the original fine-grained details.

### 3.3. Window-Guided Multi-Scale Attention Mechanism

**Figure 4:**
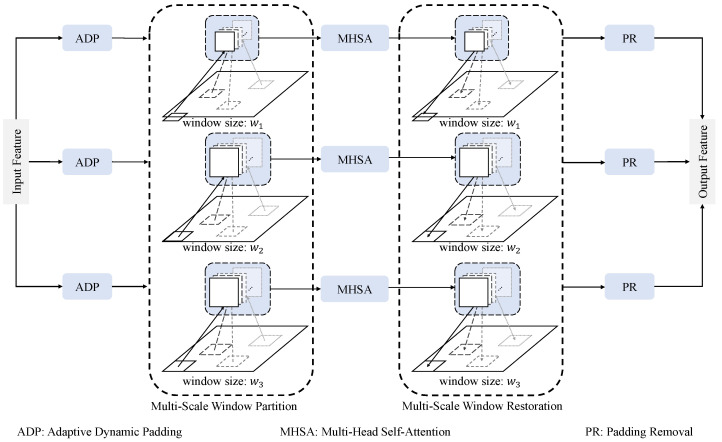
Structure of Window-Guided Multi-Scale Attention Mechanism.

This part presents the Window-Guided Multi-Scale Attention Mechanism (Figure 4) in MSGCANet, which computes the final output tensor $X_{\mathrm{out}}\in R^{B\;\times \;N\;\times \;C}$ through self-attention operations performed within localized windows of varying sizes, effectively integrating multi-scale contextual information.

The flattening operation compromises the inherent 2D spatial relationships among pixels, hindering the window mechanism’s capacity to effectively capture local contextual information. To mitigate this issue, the WGMSAM framework transforms the input feature map $X\in R^{B\;\times \;N\;\times \;C}$ (where *B* denotes batch size, N=H × W represents the spatial token count, and *H*, *W*, and *C* correspond to height, width, and channel dimensions, respectively) into a 4D tensor $X^{\prime }\in R^{B\;\times \;H\;\times \;W\;\times \;C}$, thereby reconstructing the original spatial structure.

Before performing local feature capture via shifted windows, the input feature map must undergo boundary padding to guarantee that its spatial dimensions (*H*, *W*) are exactly divisible by the current window size $w_{k}$. The multi-scale window mechanism (where $w_{k}\in \left\{w_{1},\ldots ,w_{K}\right\}$) demonstrates distinct functional specialization for contextual information capture: small windows ($w_{1}$) specialize in local fine-grained feature extraction, the large windows ($w_{K}$) establish long-range semantic dependencies, while the intermediate scales ($w_{2},\ldots ,w_{K-1}$) construct hierarchical pathways. However, this architectural design inherently invalidates conventional static padding methods or fixed input-size constraints due to its computational demands. We therefore introduce an adaptive dynamic padding strategy that computes the required padding amounts $p_{h}^{\left(k\right)}$ and $p_{w}^{\left(k\right)}$ according to the window dimensions *k*, producing the padded feature map $X_{p}^{\left(k\right)}\in R^{B\;\times \;H_{p}^{\left(k\right)}\;\times \;W_{p}^{\left(k\right)}\;\times \;C}$, with $H_{p}^{\left(k\right)}=H+p_{h}^{\left(k\right)}$ and $W_{p}^{\left(k\right)}=W+p_{w}^{\left(k\right)}$.

For the padded feature maps $X_{p}^{\left(k\right)}$ corresponding to different window scales, we implement a non-overlapping partitioning strategy based on window size $w_{k}\;\times \;w_{k}$. Specifically, we divide the feature map into $n_{h}^{\left(k\right)}=\left\lfloor H_{p}^{\left(k\right)}/w_{k}\right\rfloor$ windows along the height dimension and $n_{w}^{\left(k\right)}=\left\lfloor W_{p}^{\left(k\right)}/w_{k}\right\rfloor$ windows along the width dimension. The window dimensions $w_{k}$ directly determine the grid partitioning configuration, with larger $w_{k}$ values resulting in fewer windows ($n_{h}\;\times \;n_{w}$) and vice versa. Through this partitioning scheme, the feature map is transformed into a windowed representation $X_{w}^{\left(k\right)}\in R^{\left(B\cdot n_{h}^{\left(k\right)}\cdot n_{w}^{\left(k\right)}\right)\times \left(w_{k}\cdot w_{k}\right)\;\times \;C}$, where each $w_{k}\;\times \;w_{k}$ window preserves the complete spatial neighborhood relationships of the original feature map. Currently, the original batch dimension *B* is expanded to $B\;\times \;n_{h}^{\left(k\right)}\;\times \;n_{w}^{\left(k\right)}$, thereby allowing efficient parallel computation at the window level.

For the window features $X_{w}^{\left(k\right)}$ obtained at each window scale *k*, multi-head attention computation is performed where each independent attention head $h_{m}$ (m=1,…,M) possesses its dedicated parameter matrices $W_{m}^{Q}$, $W_{m}^{K}$, $W_{m}^{V}\in R^{d_{h}\;\times \;d_{h}}$ (where $d_{h}=C/h$ denotes the dimension per head). The input features are projected into different representation spaces through three independent linear transformation matrices $W_{m}^{Q}$, $W_{m}^{K}$, and $W_{m}^{V}$:

(5) $$\begin{array}{c} Q_{m}^{\left(k\right)}=X_{w}^{\left(k\right)}W_{m}^{Q},\;K_{m}^{\left(k\right)}=X_{w}^{\left(k\right)}W_{m}^{K},\;V_{m}^{\left(k\right)}=X_{w}^{\left(k\right)}W_{m}^{V} \end{array}$$

where for each window scale *k* and attention head $h_{m}$, its query vectors $Q_{m}^{\left(k\right)}$ actively retrieve relevant features, key vectors $K_{m}^{\left(k\right)}$ establish correspondences, and value vectors $V_{m}^{\left(k\right)}$ preserve the original representations.

Subsequently, the computation proceeds with matrix multiplication between the query vectors $Q_{m}^{\left(k\right)}$ and the transposed key vectors ${K_{m}^{\left(k\right)}}^{T}$, yielding the raw similarity dot products $Q_{m}^{\left(k\right)}{K_{m}^{\left(k\right)}}^{T}$ that capture pairwise affinities between all spatial positions within each attention head. The final normalized attention weights $A_{m}^{\left(k\right)}$ are computed as:

(6) $$A_{m}^{\left(k\right)}=\mathrm{softmax}\left(\frac{Q_{m}^{\left(k\right)}{K_{m}^{\left(k\right)}}^{T}}{\sqrt{d_{h}}}+R_{\mathrm{rel}}^{k}\right)$$

where each element aij quantifies the dynamic association strength between query position *i* and key position *j*, satisfying the probability normalization condition $\sum_{j}ai\;j=1$. This formulation establishes an adaptive feature aggregation mechanism within the local window.

Notably, we further incorporate window-wise relative position encoding $R{\mathrm{rel}}^{k}$ to inject geometric structural information, where the encoding’s coordinate range dynamically adapts with window size $w_{k}$. This encoding is implemented through a learnable relative position bias matrix $B\in R^{\left(2w_{k}-1\right)\;\times \;\left(2w_{k}-1\right)}$. For any two positions $i=(i_{x},i_{y})$ and $j=(j_{x},j_{y})$ within the window, their relative displacement $\left(\Delta x,\Delta y\right)=\left(i_{x}-j_{x}+w_{k}-1,i_{y}-j_{y}+w_{k}-1\right)$ serves as indices to retrieve the corresponding bias term bΔx,Δy from B. This parameterization strictly guarantees translation equivariance - when the input features undergo translation, the attention weights adapt according to relative positional relationships while preserving their fundamental capacity for geometric structure modeling. In implementation, we initialize the bias matrix using a truncated normal distribution and enable parameter sharing across different window sizes through bilinear interpolation, allowing the model to adaptively handle multi-scale features without retraining position encoding parameters.

Compared with alternative position encoding schemes, our window-relative position encoding demonstrates distinct advantages: while absolute position encoding captures global positional information, it violates the translation invariance that is fundamental to dense prediction tasks; whereas rotary position encoding (RoPE) fails to leverage its long-sequence advantages in local window scenarios. In contrast, our approach directly models local geometric relationships while maintaining translation equivariance. This design enables the attention matrix to significantly enhance the model’s perception of regular spatial patterns.

The attention weights are applied to perform a weighted summation of the value vectors, yielding the computed results $T_{m}$ (m=1,…,M) for each attention head within the window features.

(7) $$T_{m}=A_{m}^{\left(k\right)}V_{m}^{\left(k\right)},\left(m=1,\ldots ,M\right)$$

The outputs from all attention heads are then concatenated along the channel dimension to obtain the enhanced window features ${X^{\left(k\right)}}_{w}^{\prime }$ at the corresponding scale. This enables each window feature to integrate enriched information captured through diverse attention patterns across multiple heads.

Subsequently, the resultant features undergo inverse transformation processing to restore the original spatial dimensions, thereby maintaining dimensional consistency with the input features and completing the feature computation pipeline for a single window scale. By performing average aggregation on the computation results from different window scales, the final output $X_{out}\in R^{B\;\times \;N\;\times \;C}$ effectively integrates multi-scale contextual information, achieving an optimal balance between local details and global structures.

### 3.4. Cross-Level Transformer Decoder

Based on the Window-Guided Multi-Scale Attention Mechanism, MSGCANet proposes a cross-level Transformer decoder built upon hybrid multi-scale window contextual attention.

For the multi-scale feature maps ${\left\{F_{i}\right\}}_{i=1}^{4}\in R^{B\;\times \;C_{i}\;\times \;H_{i}\;\times \;W_{i}}$ extracted by the backbone and enhanced by the Receptive Field Block (RFB), we first project each level into a standardized feature space: The features with varying channel dimensions $C_{i}$ are mapped to a unified embedding space ($C_{embed}=256$), followed by batch normalization and ReLU activation. Spatial alignment is then performed by resampling all features to the base resolution $\left(H_{1},W_{1}\right)$ of the highest-resolution $F_{1}$. This preserves shallow-level fine-grained details while ensuring precise registration of deep semantic features on the high-resolution grid.

Multi-level features are projected into a unified representation space through standardized normalization and alignment. We fuse these features via arithmetic averaging:

(8) $$X=\frac{1}{L}\sum_{i=1}^{L}{\bar{F}}_{i}$$

The unified tensor X preserves alignment with the highest-resolution input: shallow features retain fine local details, while upsampled deep features provide global contextual information through their superpixel representations.

Then, the fused feature tensor $X\in R^{B\;\times \;C\;\times \;H\;\times \;W}$ is directly fed into a series of *L* cascaded Transformer blocks for iterative refinement, where the output of each block serves as input to the next:

(9) $$X^{\left(l\right)}={\mathrm{Block}}_{l}\left(X^{\left(l-1\right)}\right),\;l=1,\ldots ,L$$

For an individual block, the input feature $X^{\left(l-1\right)}\in R^{B\;\times \;D\;\times \;H\;\times \;W}$ is first flattened into a sequence $X_{\mathrm{flat}}^{\left(l-1\right)}\in R^{B\;\times \;N\;\times \;D}$ (N=HW), followed by layer normalization (LayerNorm) with mean μ and standard deviation σ computed along the channel dimension. Subsequently, spatial feature interaction is implemented through the Window-Guided Multi-Scale Attention Mechanism (WGMSAM, see Section 3.3). Multi-scale windows operate in parallel across feature levels, achieving implicit specialization through parameter differentiation of attention heads. The final output $X_{\mathrm{out}}\in R^{B\;\times \;(H\cdot W)\;\times \;C}$ integrates computational results from all window scales. As this output is in sequential form, tensor reshaping is required to restore spatial structure, followed by fusion with the input $X^{\left(l-1\right)}\in R^{B\;\times \;C\;\times \;H\;\times \;W}$ through residual connection:

(10) $$X^{\prime }\left(l-1\right)=X^{\left(l-1\right)}+\mathrm{Reshape}\left(X_{\mathrm{out}}\right)$$

To further enhance feature representation capability, layer normalization is first applied to $X^{\prime }\left(l-1\right)$, followed by feature transformation through a channel-mixing MLP. The final output $X^{\prime \prime }\left(l-1\right)$ is generated by combining the MLP output with the original features through a residual connection, which simultaneously preserves spatial dependencies and enhances semantic representation capability.

The block output $X^{\prime \prime }\left(l-1\right)$ propagates as input $X^{\left(l\right)}$ to subsequent blocks, enabling iterative refinement through cascaded processing: shallow blocks capture local details via small-window attention, while deep blocks model global semantics through large-window attention, with multi-scale window attention implicitly encoding hierarchical interactions via parameter sharing. The final output $X^{\left(L\right)}\in R^{B\;\times \;C\;\times \;H\;\times \;W}$ integrates cross-level features for joint detail-semantic representation.

## 4. Experiment

### 4.1. Dataset

**Figure 5:**
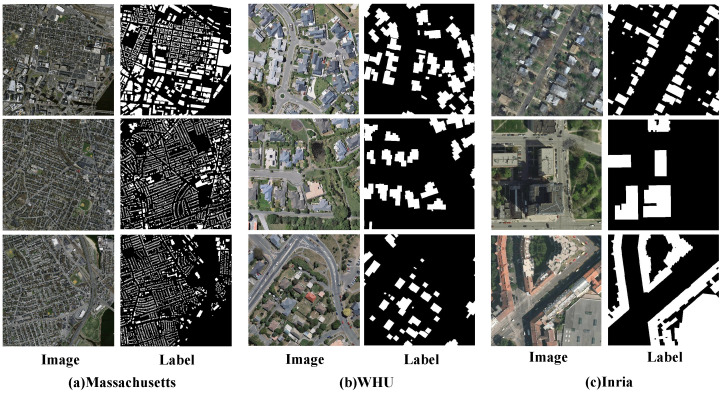
Examples of images and corresponding labels in the dataset.

For experimental validation, we employ three publicly available building extraction datasets: the WHU Building Dataset, the Massachusetts Buildings Dataset, and the Inria Building Dataset. The detailed specifications of each dataset are described below:

1. The Massachusetts Buildings Dataset [57] encompasses 151 aerial images of the Boston region. Each image measures 1500×1500 pixels, covering 2.25 km^2^ at 1-meter spatial resolution, with total coverage approximating 340 km^2^. Original partitioning designates 137 images for training, 10 for testing, and 4 for validation. Experimental preprocessing involved random cropping of images and corresponding labels to 512×512 pixel patches during training, while validation and testing employed 1536×1536 pixel padding to ensure 32-divisibility. Padded regions were systematically omitted from evaluation metrics to preserve assessment accuracy (as illustrated in Figure 5a).
2. The WHU Building Dataset [56] incorporates both aerial and satellite imagery subsets. This investigation specifically focuses on the aerial imagery subset acquired in Christchurch, New Zealand. Spanning 450 square kilometers, the subset contains annotations for over 220,000 distinct buildings extracted from source imagery with 0.075-m spatial resolution. The processed dataset comprises 8189 image tiles at 0.3-m resolution, allocated as follows: 4736 for training, 1036 for validation, and 2416 for testing (as illustrated in Figure 5b).
3. The Inria Building Dataset [58] incorporates 360 orthorectified color aerial images at 0.3-m spatial resolution, encompassing five representative urban zones in the United States (Austin, Chicago, Kitsap) and Europe (Tyrol, Vienna) with aggregate coverage of 810 km^2^ (equally allocated as 405 km^2^ per training and test set). Following official partitioning protocols, this investigation employed stratified sampling by randomly designating 1 to 5 images per city for validation while allocating residual images for training. Data preprocessing was initiated with zero-padding of original 5000×5000 pixel images to 5120×5120 pixels, subsequently segmented into standardized 512 × 512 pixel patches. Post rigorous quality control eliminated non-building specimens, and the refined dataset contained 9737 training samples and 1942 validation samples (as illustrated in Figure 5c).

### 4.2. Evaluation Metrics

To comprehensively evaluate the performance of MSGCANet, we employed four key metrics including Intersection over Union (IoU) [59], Precision (P) [60], Recall (R) [60], and F1-score (F1) [61].

(11) $$\mathrm{IoU}=\frac{TP}{TP+FP+FN}$$

(12) $$P=\frac{TP}{TP+FP}$$

(13) $$R=\frac{TP}{TP+FN}$$

(14) $$F1=\frac{2\;\times \;P\;\times \;R}{P+R}$$

where True Positives (*TPs*) denote pixels correctly identified as buildings, False Positives (*FPs*) represent non-building pixels misclassified as buildings, False Negatives (*FNs*) correspond to undetected actual building pixels, and True Negatives (*TNs*) indicate correctly classified non-building pixels.

### 4.3. Experimental Settings

All experiments were conducted on an NVIDIA GeForce RTX 3090 GPU (24 GB memory) utilizing PyTorch 1.8.1 (CUDA 11.1) to comprehensively evaluate model performance. The training protocol integrated three critical components: AdamW optimization [62] with cosine learning rate scheduling, data augmentation via random horizontal and vertical flipping, and dataset-specific parameter configurations. Following the parameter configuration in [34], our experimental setup was specified as follows: the WHU dataset employed an initial learning rate of $10^{-3}$ with a batch size of 12, the Massachusetts dataset used a learning rate of $5\;\times \;10^{-4}$ with a batch size of 2, while the Inria dataset maintained the same $5\;\times \;10^{-4}$ learning rate as Massachusetts but adopted a batch size of 12.

All experiments were conducted under the same environment and repeated five times to calculate confidence intervals, ensuring the robustness and reliability of the results.

### 4.4. Compared Methods

For a comprehensive and objective comparison, this study selects ten representative methods for performance benchmarking against MSGCANet. We employ Deeplab v3+ [21] with its atrous convolution-based feature refinement as a baseline method for general semantic segmentation. For building extraction specifically, the selected state-of-the-art approaches include CBRNet [47], ensuring segmentation consistency through contextual modeling, BuildFormer [34] and BOMSC-Net [25] utilizing Transformer architecture and multi-scale feature fusion, respectively, CLGFF-Net [36] combining a convolutional and a Transformer branch, along with DFF-Net [27] optimizing boundary recognition through dynamic feature filtering, and CICF-Net [26] employing cross-modal interaction.

As quantitatively demonstrated in Table 1, Table 2 and Table 3 through evaluation metrics including Intersection over Union (IoU), F1-score, Precision, and Recall, we systematically compare the performance disparities among these methods, with particular emphasis on their capabilities in handling multi-scale building structures and intra-class variations.

### 4.5. Evaluation on Massachusetts Building Dataset

**Figure 6:**
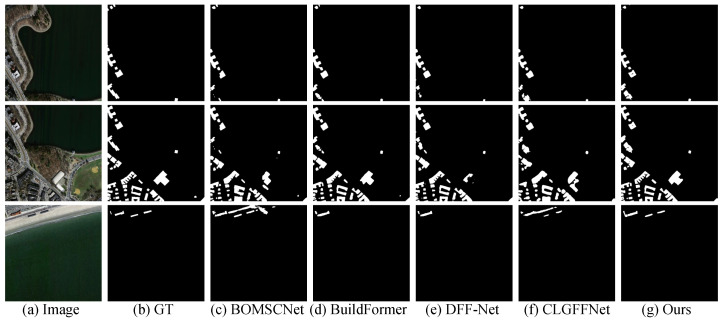
Visual comparison of the Massachusetts Building Dataset.

- Quantitative Comparison: As shown in Table 1, MSGCANet demonstrates excellent building extraction performance on the Massachusetts dataset. Among the key evaluation metrics, MSGCANet surpasses all compared methods in IoU, F1-score, and Precision, achieving 75.47%, 86.03%, and 87.55%, respectively. Its Recall reaches 84.50%, showing a clear overall advantage and reflecting the effectiveness of the multi-scale collaborative mechanism in building extraction tasks.
- Visual Comparison: Figure 6 presents three sets of visual comparisons for building extraction. As shown, in the first row, other methods miss buildings within the red-boxed regions and produce incomplete building contours, whereas MSGCANet accurately and completely extracts the building outlines, closely matching the ground truth. In the second row, the T-shaped building in the red box is incompletely captured by other methods, while MSGCANet produces contours nearly identical to the ground truth. In the third row, for the elongated buildings within the red-boxed area, BOMSC-Net and CLGFF-Net exhibit over-detection errors, and BuildFormer and DFF-Net miss certain buildings. Only MSGCANet successfully extracts the building group accurately and completely, with minimal deviation from the ground truth. These results demonstrate MSGCANet’s superiority in preserving structural details and maintaining complete building contours.

### 4.6. Evaluation on WHU Building Dataset

**Figure 7:**
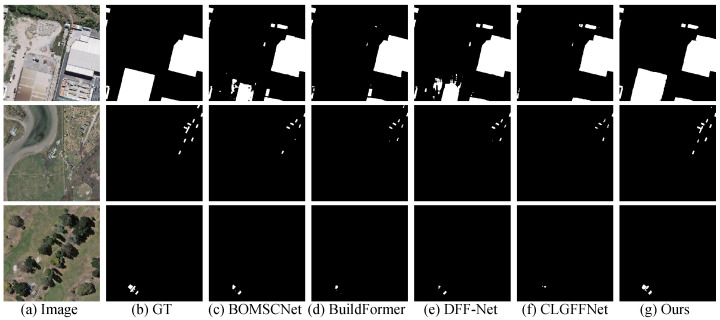
Visual comparison of the WHU Building Dataset.

- Quantitative Comparison: As shown in Table 2, MSGCANet demonstrates outstanding building extraction performance on the WHU dataset. In several key evaluation metrics, MSGCANet outperforms all compared methods, achieving an IoU of 91.53%, an F1-score of 95.59%, and a Precision of 95.65%. Additionally, with a Recall of 95.46%, MSGCANet shows a clear overall advantage, highlighting the effectiveness of the multi-scale collaborative mechanism in building extraction tasks.
- Visual Comparison: To more intuitively demonstrate the advantages of MSGCANet, Figure 7 presents the building extraction results of various comparative methods. In the first image, the building region in the lower-left corner, highlighted by a red box, suffers from missed detections in all other methods, with incomplete or entirely undetected contours. MSGCANet, however, successfully extracts complete and accurate building contours, showing high consistency with the ground truth. In the second image, the three small buildings highlighted by a red box are incompletely detected by other methods, while MSGCANet accurately captures both the number and contours of the buildings, detecting all three completely. In the third image, the dense building cluster in the lower-right corner is partially missed by other methods, resulting in incomplete extraction, whereas MSGCANet achieves complete extraction with results closely aligned with the ground truth, demonstrating very high accuracy.

### 4.7. Evaluation on Inria Building Dataset

**Figure 8:**
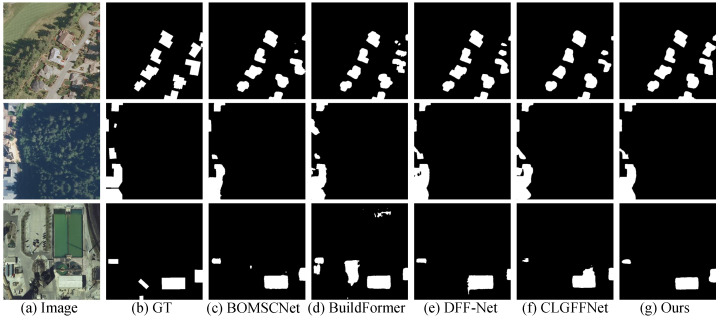
Visual comparison of the Inria Building Dataset.

- Quantitative Comparison: Table 3 presents a performance comparison of various methods on the Inria dataset. MSGCANet outperforms all other methods across all key metrics, achieving an IoU of 83.10%, an F1-score of 90.78%, a Precision of 91.98%, and a Recall of 89.55%. This demonstrates its clear overall advantage and highlights the effectiveness of the multi-scale collaborative mechanism in building extraction tasks.
- Visual Comparison: Figure 8 presents three representative cases. In the first image, the building highlighted by the red box is incompletely detected by all other methods, whereas MSGCANet successfully extracts the full building contour with high accuracy, closely matching the ground truth. In the second image, the buildings within the red box show noticeable contour deviations and shape distortions in other methods, while MSGCANet achieves the most accurate and faithful representation of the building shapes. In the third image, the small building in the lower-left corner of the red box is not perfectly detected by any method, including ours; however, the large building on the right is extracted with the most complete and precise contours by MSGCANet, demonstrating overall superior performance compared to the other methods.

## 5. Discussion

In this section, we conduct comprehensive experiments on three building datasets to validate the effectiveness of our proposed key components. Using PVTv2 as the encoder combined with a conventional decoding strategy as our baseline model, we systematically evaluate the contributions of CEM and WGMSAM. Furthermore, we specifically examine the performance variations under different window configurations in WGMSAM.

### 5.1. Effectiveness of Contextual Exploration Module

Building upon the feature representations extracted by the PVTv2 encoder, CEM employs a parallel multi-branch architecture to achieve multi-level feature enhancement. As quantitatively demonstrated in Table 4, the CEM-enhanced model exhibits statistically significant improvements in extraction accuracy metrics across all three standard building extraction datasets compared to the baseline model.

To evaluate the effectiveness of our proposed modules, we conducted statistical significance tests based on five independent trials for each configuration on the WHU, Massachusetts, and Inria datasets. The full model (baseline + CEM + WGMSAM) consistently achieves the best performance with narrow confidence intervals, indicating stable and reliable improvements. Both the CEM and WGMSAM modules contribute positively when applied individually, as evidenced by their increased IoU and F1 metrics relative to the baseline. The integration of both modules leads to further gains, with the combined model outperforming all partial configurations across all datasets. Statistical tests confirm that these improvements are significant, highlighting the complementary strengths of the CEM and WGMSAM modules and validating the effectiveness of contextual and attention mechanisms in enhancing building extraction accuracy.

**Figure 9:**
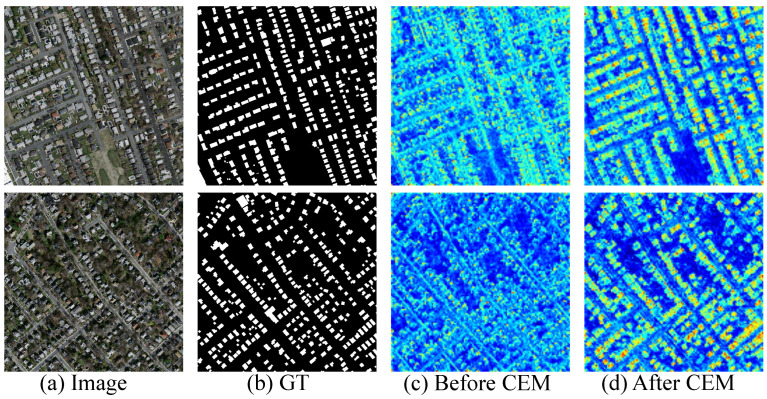
Feature visualization before/after the Contextual Exploration Module.

Visual feature analysis in Figure 9 further reveals that in CEM-augmented feature maps, the highlighted regions corresponding to building areas show substantially improved spatial alignment with ground truth annotations, while the feature discriminability between buildings and background is markedly enhanced. This improvement manifests as increased inter-class dispersion and intra-class compactness in the feature map’s color distribution. These collective advancements validate CEM’s dual advantages in enhancing feature discriminability while preserving geometric details, particularly demonstrating its critical role in achieving precise boundary delineation of buildings in complex scenes.

### 5.2. Effectiveness of Window-Guided Multi-Scale Attention Mechanism

The WGMSAM module represents the core algorithmic innovation of MSGCANet, with its exceptional performance in building extraction tasks being clearly demonstrated in Table 4. This novel multi-scale window attention mechanism achieves significant performance improvements across three benchmark datasets. Our comparative analysis reveals that while the standalone implementation of WGMSAM already shows substantial gains in both IoU and F1-score metrics, its synergistic integration with the CEM module yields even more pronounced performance enhancements.

To rigorously validate these improvements, we conducted statistical significance tests over five independent runs. The full model (baseline + CEM + WGMSAM) consistently achieves the best results with narrow confidence intervals. Paired t-tests confirm that the increases in both IoU and F1 metrics are statistically significant compared to the baseline and single-module configurations, underscoring the robustness and complementary nature of combining CEM with WGMSAM.

These results provide compelling evidence for the superior capability of WGMSAM’s multi-scale window attention mechanism in facilitating cross-window interactions and dynamic receptive field control, and the effective complementary relationship between the multi-scale attention mechanism and the context exploration module.

### 5.3. Analysis About the Windows of Window-Guided Multi-Scale Attention Mechanism

We conducted a comprehensive comparative analysis of different window configuration schemes in the WGMSAM module across three building extraction datasets in Table 5. The quantitative results for single-window configurations reveal distinct performance patterns: (1) small windows (2 × 2) achieve superior performance on the WHU dataset compared to Massachusetts and Inria, demonstrating their particular efficacy for high-resolution building clusters; (2) medium windows (4 × 4) show optimal performance on Inria, validating their adaptability to complex urban architectures; (3) large windows (8 × 8) consistently attain the highest single-window performance across all datasets, underscoring the critical importance of global context capture.

Statistical analysis over five independent trials confirms that the multi-window configuration combining all three scales (2,4,8) consistently achieves the highest IoU and F1-scores across all datasets. This configuration shows statistically significant improvements compared to all single- and dual-window variants, supported by narrow confidence intervals. These findings underscore the robustness and effectiveness of multi-scale context integration in improving building extraction accuracy.

**Figure 10:**
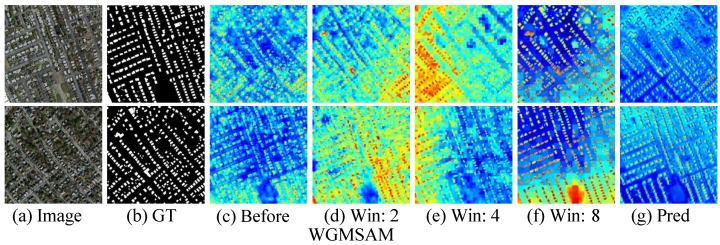
Feature visualization of the output of the WGMSAM.

Feature map visualizations (Figure 10) further illustrate these findings: Small windows generate discrete activation points that precisely align with individual building contours in ground truth annotations, though with occasional local omissions. Medium windows produce block-like activation regions that effectively capture spatial relationships among medium-sized building clusters, albeit with minor background noise incorporation. Large windows create extensive high-activation zones that comprehensively cover building group layouts, albeit with increased edge blurring and reduced boundary sharpness compared to ground truth.

The multi-window configuration demonstrates significant improvements over single-window setups. Specifically, (1) the 2 × 2+8 × 8 combination achieves optimal performance on WHU and Inria datasets, while (2) the 4 × 4+8 × 8 configuration shows superior results on Massachusetts, with both cases robustly validating the complementary nature of local-global window scales. Most notably, the triple-window combination delivers comprehensively optimal performance, exhibiting measurable gains over both the best single-window and dual-window configurations, thereby conclusively confirming the necessity of multi-scale collaboration.

### 5.4. Limitations and Future Work

While the proposed method has achieved significant progress in building extraction tasks, several noteworthy limitations warrant further investigation. At the feature representation level, the current model demonstrates limited capability in capturing vertical structural characteristics of super-tall buildings, primarily due to the inherent constraints of 2D convolutional neural networks in modeling three-dimensional spatial information. Particularly in dense urban scenarios with skyscraper clusters, the coupling relationship between complex façade reflectance properties and rooftop features remains insufficiently modeled. Furthermore, in areas with severe shadow occlusion, the segmentation accuracy still exhibits approximately 15% relative degradation, which is directly attributable to local feature distortion induced by shadows.

To address these limitations, we propose to develop a synergistic architecture incorporating 3D convolutional branches and deformable attention mechanisms to enhance the representation of building volumetric structures. Additionally, we plan to implement physics-based rendering algorithms for shadow generation, which will simulate illumination conditions at various solar elevation angles to construct more challenging training samples. These two directions constitute our primary improvement strategies.

Notably, the core concept of multi-scale contextual cooperative modeling in our approach can be extended to other geospatial extraction tasks with significant scale variations, such as road network extraction and farmland boundary detection. This provides valuable insights for developing a universal framework for remote sensing image interpretation.

## 6. Conclusions

This study addresses the critical challenges of intra-class variability and multi-scale distribution characteristics in high-resolution remote sensing image building extraction tasks by proposing a Transformer-based Multi-Scale Guided Context-Aware Network (MSGCANet). First, we construct a hierarchical receptive field expansion mechanism (CEM) based on asymmetric and progressive dilated convolutions, which significantly enhances contextual representation capabilities for dense prediction tasks through dynamic multi-scale feature fusion and residual-guided optimization. Second, we innovatively propose a dynamically adjustable multi-scale feature fusion method (WGMSAM), which establishes explicit hierarchical attention fields to achieve adaptive cross-scale feature aggregation while preserving local geometric constraints. Furthermore, we design a Transformer-based cross-level decoder architecture that utilizes deformable convolutions for spatial-adaptive alignment of multi-level semantic features, coupled with joint channel-spatial modeling for dual optimization. Extensive experiments on WHU, Massachusetts, and Inria datasets demonstrate that MSGCANet significantly outperforms state-of-the-art methods in edge integrity and small-target detection accuracy, validating its superior generalization capability in complex scenarios.

Although the dynamic window partitioning strategy effectively establishes cross-scale spatial dependencies, the adaptive determination of optimal combination weights for multi-scale windows remains an unresolved challenge. Particularly for building clusters with significant scale variations, fixed-ratio window combinations may inadequately adapt to scene characteristics. Future research could focus on developing scene-aware dynamic scale adaptation mechanisms, potentially through lightweight gating networks, to achieve adaptive allocation of window weights, thereby further enhancing the model’s scale adaptability in complex architectural scenarios. 

## Figures and Tables

**Table 1:** Performance comparison on the Massachusetts dataset. The best values in each metric are highlighted in **bold**.

Method	Year	IoU(%)	F1(%)	Pre(%)	Rec(%)
Deeplab v3+	2018	69.90	82.28	83.81	80.81
CBRNet	2021	74.55	85.42	86.50	84.36
BuildFormer	2022	75.03	85.73	86.69	84.79
BOMSC-Net	2022	74.71	85.13	86.64	83.68
CLGFF-Net	2024	75.33	85.93	85.03	**86.85**
DFF-Net	2024	72.60	84.20	87.20	81.30
CICF-Net	2024	75.17	85.83	-	-
**MSGCANet**	-	**75.47 ± 0.014**	**86.03 ± 0.012**	**87.55 ± 0.015**	84.50 ± 0.013

**Table 2:** Performance comparison on the WHU dataset. The best values for each metric are highlighted in **bold**.

Method	Year	IoU(%)	F1(%)	Pre(%)	Rec(%)
Deeplab v3+	2018	86.63	93.39	92.91	93.88
CBRNet	2021	91.40	95.51	95.31	95.70
BuildFormer	2022	90.73	95.14	95.15	95.14
BOMSC-Net	2022	90.15	94.80	95.14	94.50
CLGFF-Net	2024	91.30	95.45	95.01	**95.89**
DFF-Net	2024	90.50	95.00	95.40	94.60
CICF-Net	2024	91.45	95.53	-	-
**MSGCANet**	-	**91.53 ± 0.013**	**95.59 ± 0.012**	**95.65 ± 0.015**	95.46 ± 0.014

**Table 3:** Performance comparison on the Inria dataset. The best values for each metric are highlighted in **bold**.

Method	Year	IoU(%)	F1(%)	Pre(%)	Rec(%)
Deeplab v3+	2018	76.80	86.88	87.35	86.40
CBRNet	2021	81.10	89.56	89.93	89.20
BuildFormer	2022	81.24	89.71	90.65	88.78
BOMSC-Net	2022	78.18	87.75	87.93	87.58
CLGFF-Net	2024	82.48	90.40	91.86	88.99
DFF-Net	2024	77.90	87.60	88.80	86.30
CICF-Net	2024	81.28	89.67	-	-
**MSGCANet**	-	**83.10 ± 0.015**	**90.78 ± 0.012**	**91.98 ± 0.017**	**89.55 ± 0.013**

**Table 4:** Ablation study results on the test datasets are reported as mean ± standard deviation over five independent runs to indicate variability, along with 95% confidence intervals to assess the reliability of the improvements. Statistical significance tests were conducted to validate the results. A, B, and C denote the baseline, CEM module, and WGMSAM module, respectively.

Config			WHU		Mass		Inria	
A	B	C	IoU↑	F1↑	IoU↑	F1↑	IoU↑	F1↑
✓			88.40 ± 0.021	93.97 ± 0.018	72.51 ± 0.025	84.13 ± 0.017	78.42 ± 0.019	87.95 ± 0.022
✓	✓		90.61 ± 0.020	95.08 ± 0.017	73.75 ± 0.019	85.25 ± 0.023	80.40 ± 0.015	89.06 ± 0.027
✓		✓	91.18 ± 0.026	95.36 ± 0.022	74.66 ± 0.018	85.07 ± 0.026	82.19 ± 0.021	90.11 ± 0.025
✓	✓	✓	**91.55 ± 0.016**	**95.59 ± 0.018**	**75.45 ± 0.027**	**86.03 ± 0.020**	**83.11 ± 0.015**	**90.77 ± 0.017**

Note: The symbols indicate: ↑ higher value is better, **bold** best performance, ✓ module enabled.

**Table 5:** Ablation study results on the test datasets are reported as mean ± standard deviation over five independent runs to indicate variability, along with 95% confidence intervals to assess the reliability of the improvements. Statistical significance tests were conducted to validate the results.

Scales	WHU		Mass		Inria	
	IoU↑	F1↑	IoU↑	F1↑	IoU↑	F1↑
2	90.52±0.015	94.98±0.020	73.70±0.018	85.10±0.025	79.98±0.017	88.79±0.016
4	90.83±0.012	95.08±0.014	73.50±0.020	85.05±0.022	80.40±0.021	89.01±0.023
8	90.97±0.018	95.32±0.010	73.55±0.011	85.11±0.019	80.88±0.020	89.29±0.014
2, 4	91.21±0.020	95.40±0.025	74.70±0.022	85.62±0.021	82.30±0.019	89.88±0.015
2, 8	91.45±0.025	95.52±0.018	74.95±0.030	85.65±0.016	82.65±0.023	90.11±0.022
4, 8	91.40±0.022	95.41±0.019	75.12±0.027	85.77±0.021	82.50±0.020	90.05±0.018
2, 4, 8	91.57±0.028	95.59±0.023	75.34±0.025	85.92±0.027	83.02±0.026	90.68±0.021

Note: The symbols indicate: ↑ higher value is better, **bold** best performance.

## Data Availability

The data will be made available on request.
